# Rapid progression of recurrent intracranial solitary fibrous tumor following a previously reported TERT-wild-type primary tumor: a case report

**DOI:** 10.3389/fonc.2026.1925221

**Published:** 2026-09-08

**Authors:** Longfei Shao, Chao Yang, Xukun Teng, Jianmin Yang, Jinyang Li, Yinghao Gu, Shuo Sun

**Affiliations:** 1 School of Clinical Medicine Shandong Second Medical University, Weifang, China; 2 Jinan City Zhangqiu District People’s Hospital, Jinan, China; 3 Department of Neurosurgery, Zibo Central Hospital, Zibo, China

**Keywords:** intracranial solitary fibrous tumor, Ki-67, malignant progression, risk stratification, STAT6, WHO grading

## Abstract

**Background:**

Intracranial solitary fibrous tumor (SFT) is a rare mesenchymal neoplasm. Malignant progression of low-grade SFT typically occurs over an extended time course and in the context of TERT promoter mutations. Rapid progression with marked proliferative escalation following a previously reported TERT-wild-type primary tumor is exceptionally rare.

**Case presentation:**

A 70-year-old male had undergone resection of an anterior skull base lesion at an outside hospital 12 months prior. The outside pathology report diagnosed CNS WHO grade 2 intracranial SFT, describing 2–3 mitotic figures/10 HPF with small foci of necrosis, STAT6 nuclear positivity, a Ki-67 index of approximately 15%, and wild-type TERT promoter status. At our institution, the patient presented with left-sided limb weakness and facial deviation. Preoperative imaging was interpreted as recurrent meningioma. Gross total resection (Simpson grade I) was performed. Histopathology of the recurrent tumor revealed a hypercellular spindle cell neoplasm with patternless architecture, staghorn vasculature, diffuse nuclear atypia, up to 10 mitotic figures/10 HPF, geographic necrosis, and a Ki-67 index of 70%. Diffuse nuclear STAT6 expression supported the diagnosis of recurrent SFT in the appropriate clinical and anatomical context. Notably, the original slides from the first surgery were not available for direct histopathological review at our institution; the primary tumor grading relies exclusively on the outside pathology report. Preoperative chest CT showed no evidence of thoracic metastasis; however, abdominopelvic staging was not performed.

**Conclusion:**

This case illustrates the potential for rapid clinical and proliferative deterioration of intracranial SFT following a previously reported TERT-wild-type primary tumor. The Ki-67 index serves as a core quantitative indicator of proliferative activity. Given the unavailability of original slides for independent review, the precise WHO grade of the primary tumor cannot be independently verified, which limits definitive conclusions regarding cross-grade progression. Dura-based SFT shares substantial imaging overlap with meningioma, necessitating STAT6 immunohistochemistry for accurate differential diagnosis. Individualized postoperative surveillance should be informed by validated risk stratification models.

## Introduction

Solitary fibrous tumor (SFT) is a rare mesenchymal neoplasm first described in the intracranial compartment by Carneiro et al. in 1996 (1, 2), accounting for approximately 0.09% of intracranial tumors (3). The 2021 WHO Classification of Tumors of the Central Nervous System stratifies SFT into three grades: CNS WHO grade 1 (< 2.5 mitoses/mm², equivalent to < 5 mitoses/10 HPF), CNS WHO grade 2 (≥ 2.5 mitoses/mm² without necrosis), and CNS WHO grade 3 (≥ 2.5 mitoses/mm² with necrosis) (4). The NAB2-STAT6 fusion gene is the characteristic molecular hallmark, and nuclear STAT6 expression on immunohistochemistry (IHC) has become the diagnostic gold standard (5, 6). TERT promoter mutations are recognized as a core molecular driver of malignant progression in SFT (7, 8); However, progression in the absence of TERT mutations remains poorly characterized (9). Dura-based SFTs are frequently misdiagnosed as meningiomas due to overlapping imaging features (10–12), yet the two entities diverge substantially in recurrence risk, surveillance protocols, and indications for adjuvant therapy (13). Herein, we report a case of intracranial SFT that recurred with marked proliferative escalation over a 12-month interval following a previously reported TERT-wild-type primary tumor, and discuss implications for differential diagnosis, risk stratification, and clinical management, while acknowledging the limitations inherent in reliance on outside pathology data.

## Case presentation

A 70-year-old male had undergone resection of an anterior skull base lesion at Peking University Third Hospital on May 8, 2025 (approximately 12 months prior to the current presentation). Per the outside pathology report, the diagnosis was intracranial SFT, CNS WHO grade 2, with 2–3 mitotic figures per 10 high-power fields (HPF) and small foci of necrosis. IHC demonstrated diffuse nuclear STAT6 positivity, and the Ki-67 index was approximately 15%. Molecular testing—limited to Sanger sequencing of the TERT promoter C250T and C228T loci—revealed wild-type status. No wider next-generation sequencing (NGS) panel or cytogenetic testing was performed. After reviewing the patient’s medical records from the outside hospital, it was confirmed that the extent of resection during the initial surgery was reported as gross-total resection. The patient recovered uneventfully and did not receive adjuvant radiotherapy, targeted therapy, or other adjuvant treatment. Regular follow-up was advised. Critically, original histology slides and formalin-fixed paraffin-embedded tissue blocks from the first surgery were not available for direct review at our institution; all primary tumor data presented herein are derived exclusively from the outside pathology report, and independent verification of the WHO grade, mitotic count, and Ki-67 index was therefore not possible. On May 23, 2026, the patient was admitted to the Department of Neurosurgery at Zibo Central Hospital with a two-day history of left-sided limb weakness and deviation of the angle of the mouth. He denied headache, nausea, vomiting, seizures, or altered consciousness. Past medical history was unremarkable. Physical examination revealed alert mental status with reduced left limb muscle strength (grade 4/5) and left facial palsy. Vital signs were stable. The exact surgery date was in 29 May 2026, and the last follow-up was in 20-July 2026, yielding approximately 7 weeks (52 days) of follow-up.

**Figure 1:**
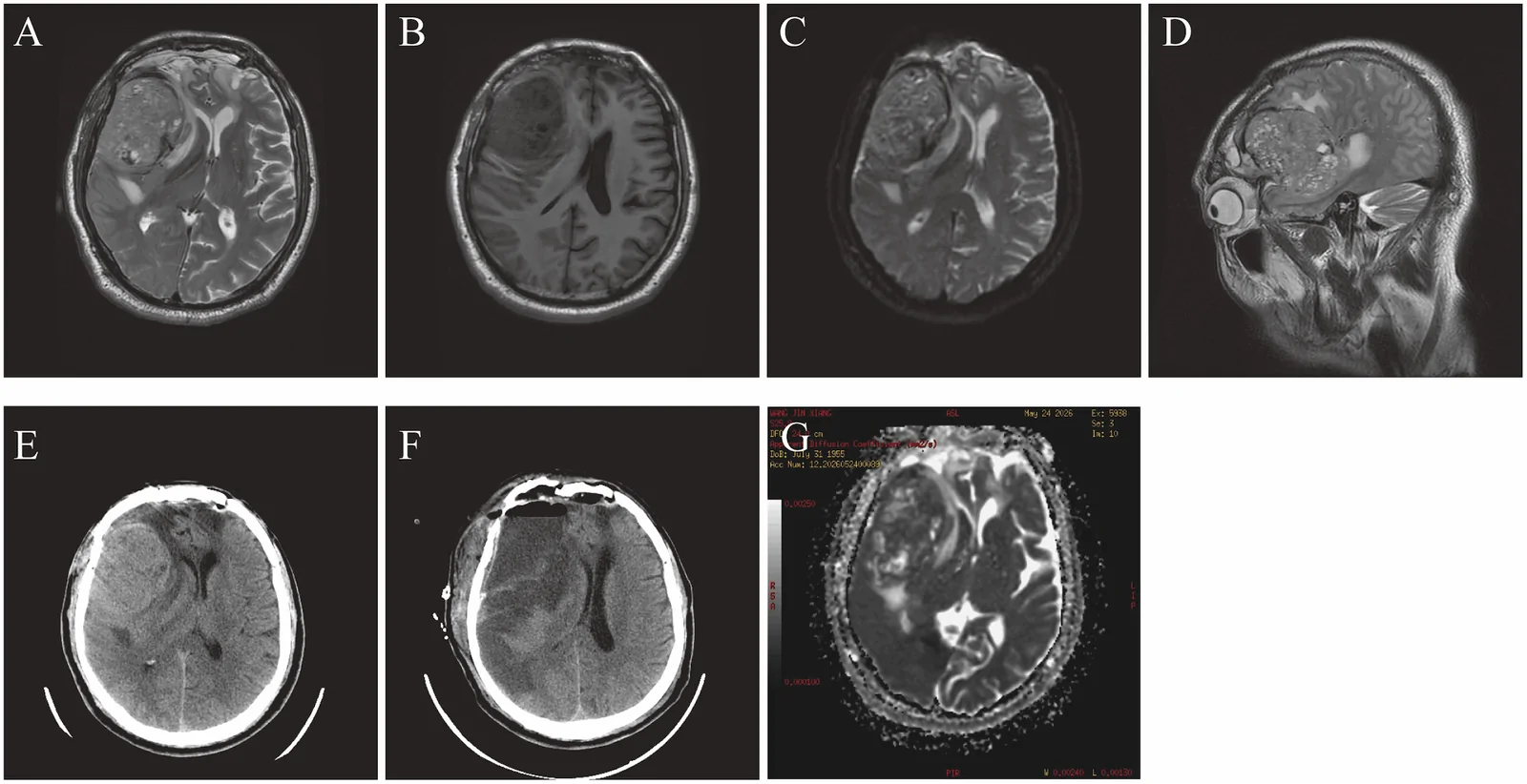
Imaging findings of recurrent intracranial SFT. **(A)** Axial T2-weighted MRI shows a mass in the right frontal-temporal lobe with extensive dural attachment, mass effect, ventricular compression, and leftward midline shift. **(B)** Contrast-enhanced T1-weighted MRI shows marked homogeneous enhancement. **(C)** Diffusion-weighted imaging (DWI) shows mixed signal intensity without obvious diffusion restriction. **(D)** Sagittal T2-weighted MRI shows the anteroposterior extent of the lesion. **(E)** Preoperative axial CT scan shows a high-density extraxial mass. **(F)** Postoperative axial CT scan confirms gross complete resection. **(G)** ADC map. The lesion was diagnosed as a recurrent meningioma based on imaging findings.

Preoperative contrast-enhanced brain MRI revealed a right frontotemporal subdural space-occupying lesion with broad dural attachment and prominent homogeneous enhancement (**Figure 1**). On T1-weighted imaging, the lesion appeared isointense to gray matter; T2-weighted sequences demonstrated heterogeneous hyperintensity with peritumoral edema. Diffusion-weighted imaging (DWI) showed mixed signal without marked restricted diffusion, and the apparent diffusion coefficient (ADC) map displayed intermediate values. A dural tail sign was present, and flow voids were not conspicuous. The lesion was initially interpreted radiologically as recurrent meningioma. Preoperative chest CT revealed no evidence of thoracic metastatic disease; abdominopelvic staging was not performed.

**Figure 2:**
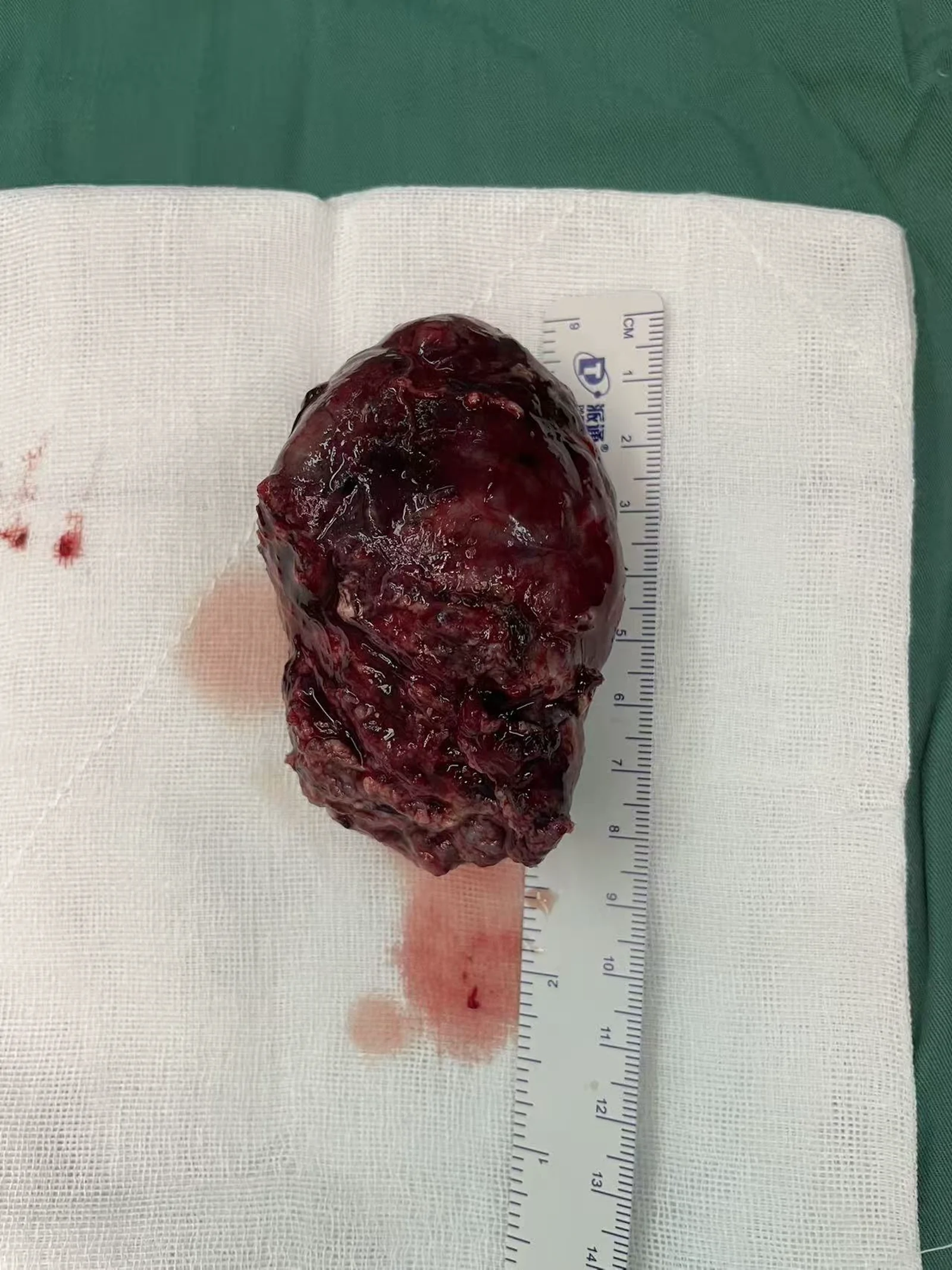
Gross specimen of the recurrent intracranial SFT. The resected tumor measures 8.0 × 5.0 × 5.0 cm with an irregular contour, prominent surface hemorrhage and focal surface necrosis are noted externally; no cut-section photograph is available. Part of the tumor is adherent to native dura mater. Scale bar: centimeter ruler.

Following routine preoperative evaluation, the patient underwent gross total resection (GTR) of the right frontotemporal lesion via craniotomy. Intraoperatively, the tumor was firm, hypervascular, and adherent to the underlying dura and adjacent brain parenchyma; complete macroscopic resection (Simpson grade I) was achieved. Postoperative contrast-enhanced MRI within 48 hours confirmed no residual enhancing tumor. Gross examination of the resected specimen revealed an irregular mass measuring 8.0 × 5.0 × 5.0 cm (**Figure 2**). The external surface of the tumor was predominantly grayish-white with multifocal areas of surface hemorrhage and apparent necrosis, markedly different from the homogeneous tan-white appearance typical of low-grade SFT. A dedicated cut-section photograph was not obtained; the gross description of the internal architecture is therefore limited to the external appearance.

**Figure 3:**
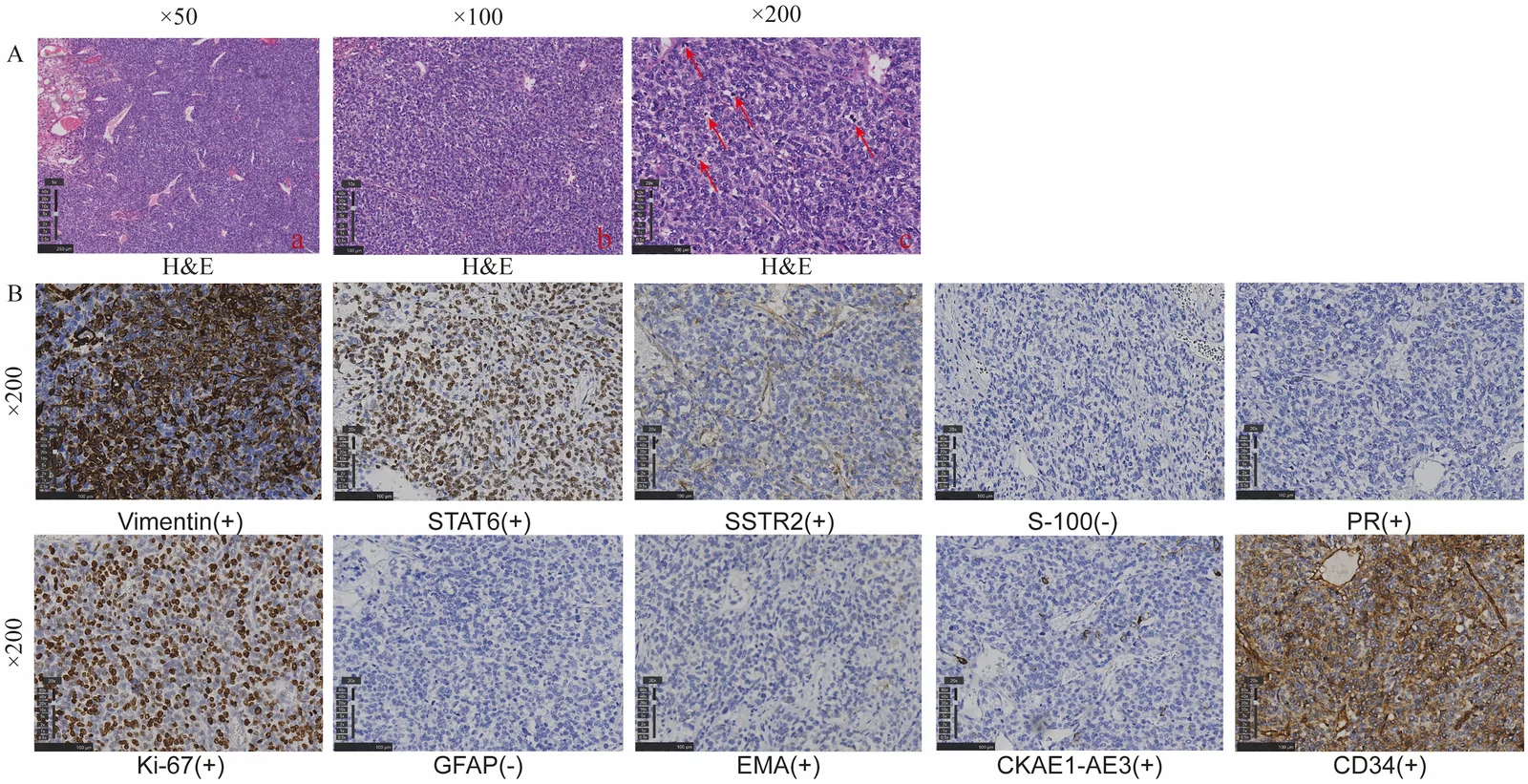
Histopathology and immunohistochemistry of the recurrent intracranial SFT. **(A)** H&E staining: (a) low-power view (×50) showing a hypercellular spindle cell neoplasm with patternless architecture, interspersed collagen bands, and prominent branching (staghorn) vasculature within brain parenchyma; (b) intermediate-power view (×100) showing tumor cells that are short fusiform to round, with sparse cytoplasm, fine chromatin, and moderate-to-severe nuclear atypia; (c) high-power view (×200) showing marked tumor cell pleomorphism; arrows indicate five mitotic figures. **(B)** Immunohistochemistry (all ×200): Vimentin diffusely positive; STAT6 showing diffuse nuclear positivity, supporting the diagnosis of SFT; SSTR2 showing focal positivity; S-100 negative in tumor cells; PR showing focal positivity; Ki-67 demonstrating a high proliferation index of approximately 70% (hotspot method, manual count of approximately 1,000 tumor cells, necrotic zones excluded); GFAP negative in tumor cells, excluding glial origin; EMA showing focal positivity; CKAE1-AE3 showing focal positivity; CD34 showing extensive positivity. Comparative primary tumor IHC images were not available for inclusion, as original slides from the first surgery could not be obtained for review.

Microscopic examination (**Figure 3**) revealed a hypercellular spindle cell neoplasm with patternless architecture, interspersed collagen bands, and prominent branching (staghorn) vasculature. Tumor cells exhibited moderate-to-severe nuclear atypia with hyperchromasia and irregular nuclear contours. Diffuse atypical proliferation was accompanied by widespread multifocal hemorrhage and geographic necrosis. Mitotic activity was markedly elevated, with up to 10 mitotic figures/10 HPF. Infiltrative margins into adjacent brain parenchyma were identified. No evidence of dedifferentiation was observed. Comparative IHC of the recurrent tumor demonstrated diffuse nuclear positivity for STAT6, confirming SFT diagnosis. The Ki-67 index was markedly elevated at 70% (hotspot method, manual count of approximately 1,000 tumor cells, necrotic zones excluded). CD34 showed partial positivity; vimentin was diffusely positive. Focal positivity was observed for EMA, PR, CKAE1/AE3, and SSTR2—markers commonly expressed in meningiomas. However, the presence of diffuse nuclear STAT6 positivity, a highly sensitive and specific surrogate for NAB2::STAT6 fusion in the appropriate morphological context (5, 6), definitively established the diagnosis of SFT and excluded meningioma, as focal expression of epithelial and meningothelial markers is recognized in SFT and does not alter the diagnosis when STAT6 is diffusely positive (13–16) It should be noted that NAB2::STAT6 fusion was not directly confirmed by molecular testing in the recurrent tumor; the diagnosis relies on the well-established surrogate value of diffuse nuclear STAT6 immunohistochemistry. Negative staining for S-100 and GFAP excluded glial and nerve sheath tumors; p53 immunohistochemistry showed a wild-type pattern. The final pathological diagnosis was SFT, CNS WHO grade 3.

Perioperative management included hemostasis, dehydration to reduce intracranial pressure, neurotrophic support, and infection prophylaxis. The patient’s left limb weakness and facial deviation gradually improved, and he was discharged with stable vital signs. Adjuvant radiotherapy was recommended given the WHO grade 3 histology; however, after a shared decision-making discussion regarding risks and benefits, the patient declined this intervention. At the most recent follow-up (approximately 7 weeks postoperatively (52 days; last follow-up: 20 July 2026)), the patient remained clinically stable without new neurological deficits. Given the high-grade pathology and elevated proliferative index, individualized intensified surveillance with contrast-enhanced brain MRI at 3- to 4-month intervals for the first two years has been proposed as an institutional practice, recognizing that a universally validated surveillance schedule for this clinical scenario is not established.

## Discussion

Large-scale case series have demonstrated that malignant progression of intracranial SFT predominantly occurs in the context of late recurrence beyond 3 years postoperatively (14, 15), and most such cases harbor TERT promoter mutations as the canonical driver of malignant progression (7, 8). The present case is notable for the exceptionally short 12-month interval between primary resection and recurrence with marked proliferative escalation, occurring in a previously reported TERT-wild-type primary tumor (**Table 1**). The recurrent lesion arose in the right frontotemporal region, anatomically distinct from the original anterior skull base location. This spatial discrepancy may reflect multicentric dural-based SFT, subclinical dural dissemination along the cerebrospinal fluid pathway or meningeal surfaces following the initial surgery, or independent clonal evolution of residual tumor cells within a different dural compartment; however, without molecular clonality analysis, the precise relationship cannot be definitively established. A systematic review of the literature indicates that the majority of reported intracranial SFT cases undergoing clinical deterioration do so over substantially longer intervals (typically ≥ 36 months) and in the presence of TERT promoter mutations (9, 16, 17).

**Table 1:** Summary of reported intracranial SFT cases with malignant progression.

Study	Age/sex	Initial grade	Recurrence grade	Interval (months)	TERT status	Outcome
Present case	70/M	WHO 2*	WHO 3	12	Wild-type	Alive, ~7 weeks FU
Vogels et al., 2019 (9)	Variable	WHO 1–2	WHO 3	24–120+	Mutant (majority)	Variable
Fritchie et al., 2019 (16)	Variable	WHO 1–2	WHO 3	36–180	Mutant/WT	Variable
Eschbacher et al., 2024 (17)	Variable	WHO 2	WHO 3	>36	Mutant predominant	Variable
Khan et al., 2024 (11)	Variable	WHO 1–2	WHO 2–3	12–96	Not reported	Variable

*The initial WHO grade is based on the outside pathology report and could not be independently verified due to unavailability of original slides. See Discussion for details.

An important interpretive caveat must be acknowledged regarding the initial WHO grade. The 2021 CNS WHO classification defines grade 2 SFT as having ≥ 2.5 mitoses/mm² (≥ 5 mitoses/10 HPF) without necrosis, and grade 3 as having ≥ 2.5 mitoses/mm² with necrosis (4). According to the outside pathology report, the primary tumor exhibited 2–3 mitotic figures/10 HPF—below the threshold of 5 mitoses/10 HPF required for WHO grade 2 or 3 designation—yet small foci of necrosis were described. By strict application of the 2021 WHO criteria, the reported mitotic count (< 5/10 HPF) would place the primary tumor in the WHO grade 1 category, making the presence of necrosis incongruent with a low-grade designation. Several factors complicate this assessment: (i) Ki-67 quantification by visual estimation (“eyeballing”) is inherently subjective, and the reported 15% index is higher than expected for a typical low-grade SFT; (ii) the outside hospital may have employed an earlier grading system or different diagnostic criteria than the current 2021 WHO scheme; (iii) small foci of necrosis could theoretically result from preoperative embolization, surgical manipulation, or sampling artifact, though no embolization was documented. Because original histology slides and tissue blocks from the first surgery were not available for independent review at our institution, the WHO grade assigned by the outside pathologist cannot be independently confirmed or refuted. We therefore present the initial diagnosis as reported, while emphasizing that the precise grade of the primary tumor remains uncertain. Regardless of the initial WHO designation, the objective parameters—a Ki-67 increase from 15% to 70%, a mitotic count escalation from 2–3/10 HPF to up to 10/10 HPF (approximately 4.2 mitoses/mm² assuming a standard field area of 0.237 mm² per HPF), and the emergence of extensive geographic necrosis—document unequivocal proliferative acceleration and clinical deterioration over a 12-month period.

The substantial increase in the Ki-67 proliferation index—from 15% to 70%, representing an approximately 4.7-fold increase—constitutes the most direct quantitative indicator of proliferative acceleration. The Ki-67 index was assessed by manual count in the area of highest labeling (hotspot method), with approximately 1,000 tumor cells counted and necrotic zones excluded. This heightened proliferative activity was paralleled by a marked escalation in mitotic figures (from 2–3/10 HPF to up to 10 mitotic figures/10 HPF), the emergence of widespread geographic necrosis, and multifocal hemorrhage (**Table 2**). The modified Demicco risk stratification model, which incorporates patient age, tumor size, mitotic index, and extent of necrosis, has been validated for predicting recurrence and metastasis in SFT (14, 15). Applying this model with extrapolation to the intracranial compartment—recognizing that the modified Demicco model was developed and validated primarily for extrameningeal SFTs—the recurrent tumor in our patient falls into the intermediate-risk category (age ≥ 55, tumor size 5–<10 cm, mitotic count ≥ 4/10 HPF, necrosis ≥ 10%), consistent with its aggressive clinical behavior. The exact extent of necrosis was estimated at ≥10% of the tumor area. Even if the initial risk profile cannot be reliably calculated due to incomplete primary tumor data, the recurrent tumor’s intermediate-risk Demicco classification underscores the need for vigilant surveillance.

**Table 2:** Comparison of histopathological parameters between primary and recurrent tumors.

Parameter	Primary tumor (outside report)	Recurrent tumor (our institution)
WHO Grade	2 (per outside report; not independently verified)	3
Mitotic count	2–3/10 HPF	Up to 10/10 HPF
Necrosis	Small foci	Widespread geographic necrosis
Hemorrhage	Not prominent	Multifocal, extensive
Ki-67 index	~15%	~70%
Cellularity	Moderate	High, diffuse
Nuclear atypia	Mild	Moderate-to-severe
Patternless architecture	Present	Present, more pronounced
Staghorn vasculature	Present	Prominent
Infiltrative margins	Not reported	Present
STAT6 IHC	Nuclear positive	Nuclear positive (diffuse)
TERT promoter (C250T/C228T)	Wild-type	Not re-tested
NGS/cytogenetics	Not performed	Not performed

Regarding molecular mechanisms, the previously reported wild-type TERT status of the primary tumor, combined with the absence of retesting in the recurrent tumor, limits definitive conclusions about the molecular drivers of progression. It should be noted that molecular characterization was limited to Sanger sequencing of the TERT promoter C250T and C228T hotspots in the primary tumor only; the recurrent tumor was not tested for TERT promoter variants, and no wider NGS panel, copy number analysis, or cytogenetic testing was performed on either specimen. We hypothesize—without direct experimental evidence—that several mechanisms may have contributed: a senescence-associated secretory phenotype (SASP) and chronic inflammatory microenvironment in the elderly host potentially activating proliferative signaling (18); clonal selection and expansion of residual malignant subclones within the surgical bed microenvironment (19); and epigenetic alterations, including aberrant DNA methylation or alternative lengthening of telomeres (ALT) (20, 21). These hypotheses remain speculative; comprehensive molecular profiling—including NAB2::STAT6 fusion subtyping, TP53, RB1, CDKN2A/B, ATRX/ALT pathway status, TERT copy number analysis, and targeted NGS—was not performed and represents a significant limitation.

Dura-based intracranial SFTs pose a substantial diagnostic challenge as they closely mimic meningiomas on contrast-enhanced MRI, exhibiting features such as the dural tail sign, homogeneous enhancement, and broad dural attachment (10–12). In the present case, the lesion was misdiagnosed radiologically as meningioma at the current presentation, several imaging features may help differentiate SFT from meningioma: SFT tends to demonstrate more heterogeneous T2 signal due to variable collagen content and cystic degeneration, more prominent flow voids reflecting hypervascularity, lobulated contours, and often lack hyperostosis or calcification that are common in meningiomas. Perfusion-weighted imaging may show higher relative cerebral blood volume in SFT (11). Nevertheless, these features are not reliably discriminatory. In our case, focal positivity for EMA, PR, CKAE1/AE3, and SSTR2—markers commonly expressed in meningiomas—initially raised diagnostic concern. However, the diffuse nuclear STAT6 positivity definitively established the diagnosis of SFT and excluded meningioma. This distinction has critical clinical implications: high-grade SFT carries a substantially higher risk of recurrence and distant metastasis compared with meningioma, necessitating different surveillance protocols and consideration of systemic therapy (13, 16).

From a clinical management perspective, gross total resection remains the cornerstone of treatment for intracranial SFT. For patients with WHO grade 3 histology or high-risk features, adjuvant radiotherapy has been associated with improved local control in retrospective series, although prospective data are lacking (22). In the present case, adjuvant radiotherapy was recommended but declined by the patient. For recurrent or metastatic SFT not amenable to further local therapy, systemic treatment options include anti-angiogenic agents such as pazopanib, which has demonstrated activity in phase 2 trials for both typical and malignant/dedifferentiated SFT (23, 24), and sunitinib, which has shown clinical benefit in retrospective and prospective series (25). Emerging molecularly targeted approaches based on NAB2::STAT6 fusion subtypes remain investigational (19, 26). The level of evidence for systemic therapies in intracranial SFT is limited, primarily derived from small series and extrapolation from extracranial SFT data.

Based on the Demicco risk model (14, 15) and the aggressive behavior observed in this case, we suggest that patients with intermediate-risk or high-risk intracranial SFT (elevated mitotic count, necrosis, large tumor size, or advanced age) undergo individualized intensified surveillance. For diagnostically challenging dura-based lesions in which meningioma is suspected but imaging features are atypical, STAT6 IHC should be performed to ensure accurate classification. For patients with intermediate-risk or high-risk features, postoperative MRI surveillance at 3- to 4-month intervals for the first two years, with progressive extension thereafter, may be considered.

## Limitations

This report has several important limitations. First, and most critically, original histology slides and formalin-fixed paraffin-embedded tissue blocks from the initial surgery at the outside hospital were not available for direct review at our institution. All data regarding the primary tumor—including WHO grade, mitotic count, Ki-67 index, and the characterization of necrosis—were derived exclusively from the outside pathology report. Consequently, independent verification of the initial WHO grade is not possible, and the possibility that the primary tumor may have already met criteria for a higher grade cannot be excluded. This uncertainty should be considered when interpreting the apparent cross-grade progression. Second, comprehensive molecular characterization—including NAB2::STAT6 fusion subtyping, TP53, RB1, CDKN2A/B, ATRX/ALT pathway status, TERT copy number analysis, copy number profiling, and targeted NGS—was not performed due to resource constraints, limiting mechanistic interpretation. Third, molecular testing of the primary tumor was limited to TERT promoter hotspot sequencing (C250T and C228T); no wider genomic or cytogenetic characterization was available. Fourth, the follow-up duration (approximately 7 weeks (52 days)) is insufficient to assess long-term outcomes. Fifth, the patient declined adjuvant radiotherapy, and therefore the impact of adjuvant treatment on local control cannot be evaluated. Sixth, abdominopelvic staging beyond chest CT was not performed, and the possibility of occult metastatic disease cannot be excluded. Despite these limitations, the comparative pathological data between primary and recurrent tumors, supported by STAT6 IHC confirmation of SFT diagnosis, provide valuable clinicopathological insights into the potential for rapid clinical deterioration of intracranial SFT.

## Conclusion

This case illustrates the potential for rapid proliferative acceleration and clinical deterioration of intracranial SFT following a previously reported TERT-wild-type primary tumor, with the Ki-67 index escalating from 15% to 70% over a 12-month interval. The precise WHO grade of the primary tumor cannot be independently verified due to unavailability of original slides, which represents the principal limitation of this report and warrants caution in drawing definitive conclusions about cross-grade progression. Nonetheless, the objective parameters document unequivocal biological worsening. Dura-based SFT shares substantial imaging overlap with meningioma, necessitating STAT6 IHC for accurate differential diagnosis. Risk stratification using validated models such as the Demicco system—with recognition of its limitations when extrapolated to intracranial SFT—should inform individualized surveillance and treatment decisions. Further studies incorporating comprehensive molecular profiling and independent histopathological review of both primary and recurrent tumors are warranted to elucidate the mechanisms underlying clinical deterioration in intracranial SFT.

## Data Availability

The original contributions presented in the study are included in the article/supplementary material. Further inquiries can be directed to the corresponding authors.
